# Prevalence and determinants of unintended pregnancy among female sex workers (FSW) in Jashore, Bangladesh

**DOI:** 10.1371/journal.pone.0342766

**Published:** 2026-02-13

**Authors:** Md. Masud Reza, Hasibul Hasan Shanto, Samira Dishti Irfan, A. K. M. Masud Rana, Mohammad Niaz Morshed Khan, Golam Sarwar, Mohammad Sha Al Imran, Mahbubur Rahman, Md. Safiullah Sarker, Muntasir Alam, Md. Abu Hena Chowdhury, Mustafizur Rahman, Sharful Islam Khan

**Affiliations:** 1 Programme for HIV and AIDS, Health Systems & Population Studies Division, International Centre for Diarrhoeal Diseases Research, Bangladesh (ICDDR,B), Dhaka, Bangladesh; 2 Virology Laboratory, Infectious Diseases Division (IDD), ICDDR,B, Dhaka, Bangladesh; 3 Department of Dermatology, Bangladesh Medical University (BMU), Dhaka, Bangladesh; Johns Hopkins Bloomberg School of Public Health, USAID/Public Health Institute, UNITED STATES OF AMERICA

## Abstract

**Background:**

Unintended pregnancy among female sex workers (FSW) is a pressing reproductive health concern attributable to risky sexual behaviors, healthcare inequities and poor negotiation powers with male sex partners. However, evidence is scarce on the prevalence and determinants of unintended pregnancies among FSW, which is crucial for enhancing reproductive healthcare. This analysis aims to measure the prevalence of lifetime unintended pregnancies and their associated factors.

**Methods:**

A cross-sectional study was conducted on 327 FSW in Jashore (a border belt district of Bangladesh) from September 2022 to March 2023. Participants were recruited through take-all sampling. Data were collected on the lifetime history of unintended pregnancies and other relevant variables through face-to-face interviews. Chi-square statistic was used to compare the characteristics of FSW reporting unintended pregnancies. To assess the net association of factors associated with unintended pregnancy, multiple logistic regression was applied.

**Result:**

The lifetime prevalence of unintended pregnancies was reported at 75.8% (95%CI: 71.0–80.1). Among those who reported unintended pregnancies, 37.1% (95%CI: 30.8–43.8) had no education, 39.9% (95%CI: 32.8–47.5) were 25–34 years old, 49.6% (95%CI: 39.3–59.9) were currently married and 62.9% (95%CI: 49.7–74.4) earned ≤10,000 BDT per month compared to those who did not report lifetime unintended pregnancies. The likelihood of unintended pregnancies was significantly higher among those who reported having sex with non-transactional male sex partners (AOR: 2.4, 95%CI: 1.1–5.3, p = 0.036) than those who never had sex with any non-transactional male sex partner. The likelihood was also higher among those who reported rape in their lifetime (AOR: 2.0, 95%CI: 1.0–3.8, p = 0.037) and who self-reported mental health problems (AOR: 2.1, 95%CI: 1.0–4.2, p = 0.045) within the past year, compared to their counterparts.

**Conclusion:**

This study highlights the considerable prevalence and associated determinants of unintended pregnancies among FSW in Jashore. These determinants need to be considered to strengthen reproductive healthcare interventions and policies for FSW. Reproductive health of FSW cannot be improved unless these factors are addressed in the ongoing interventions.

## Background

According to recent global estimates, half of the pregnancies are unintended, 60.0% of which end in abortion [1]. Unintended pregnancy is defined as a pregnancy where the woman did not plan to have any (or additional) children, or a pregnancy that occurred earlier than she planned [1–4]. Unintended pregnancies pose global health concerns due to adverse health implications such as: reduced uptake of antenatal care, unsafe abortions, maternal and neonatal mortality, and stillbirths [5–10]. They also negatively affect women’s personal and family lives, and their broader societal standing [11]. Between 2015 and 2019, approximately 121 million unintended pregnancies occurred worldwide, averaging 331,000 cases per day.

Unintended pregnancies remain a pervasive, yet unaddressed, public health challenge, particularly among underserved, marginalized groups of women such as female sex workers (FSW). Several risk factors elevate their vulnerability towards an unintended pregnancy such as unprotected sex, limited high rates of sexual abuse, and gendered power differentials which compromise their ability to negotiate safer sex with transactional and non-transactional partners [10–12]. The behavioral and structural factors associated with sex work not only contribute to unintended pregnancies but also other poor sexual and reproductive health (SRH) outcomes such as sexually transmitted infections (STIs), reproductive tract infections (RTIs), and adverse pregnancy results, such as miscarriages, abortions, and stillbirths [12–14]. Their marginality and limited access to comprehensive reproductive health services and information make addressing unintended pregnancies even more challenging [13,15,16].

In South Asia, social progress and economic development have varied widely, yet FSW in the region continue to struggle with discrimination, stigma, and legal restrictions which impede their access to sexual and reproductive health and rights (SRHR) services [17–20]. Bangladesh also faces these social, economic, and health challenges [18,21,22]. In Bangladesh, the legal status of sex work is ambiguous, leaving female sex workers with limited legal protection and exposing them to significant social stigma, thus heightening their vulnerability [18,23]. Moreover, patriarchal ideologies, enforced by spouses and in-laws, and conservative attitudes towards sexuality and reproductive health education further limit FSW’s access to contraception and healthcare services [18,24]. Beyond physical health risks [11,25], the lack of proper family planning options and support could also result in financial strain, psychological distress, and the challenges of raising children in a stigmatized environment [25]. According to the 2023 national size estimate for key populations at risk of HIV in Bangladesh, there are approximately 113,106 FSW living in Bangladesh, accounting for almost 0.24% of 46,876,305 women of reproductive age (i.e., 15–49 years) reported in the population and housing census in Bangladesh 2022 [26,27]. However, FSW bear a remarkably higher burden of unintended pregnancies. Nationally, approximately 19.0% of all pregnancies among women of reproductive age are unintended in Bangladesh [28]. Whereas, a systematic review conducted among FSW in the low- and middle-income countries (LMIC) documented an incidence of 27.1 unintended pregnancies per 100 person-years [29]. This stark contrast highlights unintended pregnancy as a uniquely pressing problem for FSW, thus justifying the importance of this study for this highly marginalized group. The existing evidence base in Bangladesh examined the patterns and determinants of unintended pregnancies among women of reproductive age [2,7,30]. Yet, this phenomenon remains under-researched among marginalized women such as FSW [11]. Given the complex and culturally sensitive context of sex work in Bangladesh, understanding the factors contributing to unintended pregnancy among FSW is critical for informing targeted reproductive healthcare interventions which can ultimately improve their SRHR outcomes. This evidence gap exists not only in Bangladesh but also in other socio-culturally similar settings with gendered power differentials and stigmatization against sex work. To address this gap, research is warranted to examine the current scenario of FSW in relation to unintended pregnancy, abortion and its consequences. In this context, this study aims to document the prevalence of unintended pregnancy and its determinants among the FSW in Jashore. Jashore was selected as the study site due to the availability of FSW and geographically well-defined cruising spots. Furthermore, the existence of non-government organizations (NGOs)/community-based organizations (CBOs) for establishing network with FSW to ensure participation for face-to-face interview was also the motivation behind choosing Jashore as the site [31]. Our study is also important to meet the SDG goal 3 (Good Health & Well-being) and target 3.7, that highlights that by 2030, ensure universal access to sexual and reproductive health-care [32].

## Methods

### Study design and data collection

This article is based on data from a cross-sectional survey of the first round of the SRHR surveillance conducted in Jashore (a border-belt district in the south-west part of Bangladesh near India) among 327 FSW from 22 September 2022 to 18 March 2023 [31]. A semi-structured questionnaire was delivered, adopting face-to-face techniques.

### Training

After recruiting team members, a comprehensive 15-day in-house training program was conducted at the head office of the host research organization in Dhaka (capital city of Bangladesh) from June 5 to June 22, 2022. The onboarded staff members included a research investigator (RI), field research officers (FRO), data management officer (DMO), data management assistant (DMA), counsellors and surveillance workers (data collectors). The training included the complexities and sensitivities of the population groups, techniques for collecting socio-economic and demographic information, issues related to reproductive health (e.g., unintended pregnancies, abortions, childbirth, contraceptive use, etc.), sexually transmitted infections (STIs), sexual dynamics of FSW and their male sex partners, available reproductive health services for FSW in Jashore, and ethical issues. This was followed by specialized training sessions on taking consent/assent, and interviewing techniques. The training leveraged the expertise of the investigators and senior members within the multidisciplinary team affiliated with the Program for HIV and AIDS, and Virology Laboratory of the institution. The training concluded with the field-testing of obtaining assent/consent and face-to-face interviews, which took place in Dhaka. Field testing experiences helped fine-tune the quality of data collection and smooth operation of the study.

### Definition of FSW

According to national documents on key populations, FSW were defined as women who sell sex and are usually contacted by or negotiate with clients at various locations such as streets, hotels, residences or brothels, in exchange of money or any other gift within the last 12 months [33]. FSW can have transactional sex and non-transactional sex. In our study, transactional sex refers to FSW who sell sex to males in exchange of money, gifts or other payments. Non-transactional sex refers to FSW who have sex with males without any monetary exchanges, usually characterize intimate partnerships [14].

### Inclusion criteria

To be eligible to participate in the first round of SRHR surveillance, FSW had to be at least 15 years old, they could not be using drugs or pregnant, and they needed to have provided written informed assent/consent.

### Ethical considerations

This study received ethical approval from the Ethical Review Committee of the Institutional Review Board of the organization on 9 April 2022. Written informed consent/assent was obtained from all participants (where assent was taken from participants aged 15–17 years old). Since sex work is deemed culturally unacceptable and legally ambiguous in Bangladesh, we could not interview FSW aged 15 to <18 years old in the presence of their legal guardians. Instead, we enlisted help from local guides (who were also 18 years or older, well-known to the participant and belonged to the same community) to identify and recruit participants. This assent-taking procedure was widely used in the previous HIV surveillance rounds in Bangladesh throughout the years [14,34–38]. The assent form was verbally read out to the guides and participants, after which their signature or thumb impression was taken.

The researchers explained the study objectives, benefits, and risks to the participants, and reassured them that their privacy and confidentiality would be maintained. Interviews were conducted in secluded rooms in the field office in Jashore where no one else was present besides the participant and interviewer. In the filled-out questionnaire, participants were assigned unique identification numbers which included particulars such as their name, location and contact details. These details were only available to the authorized research team members. Moreover, during data analysis and reporting, these participants were de-identified.

### Sample size calculation

To conduct the first round of SRHR surveillance, a list of outcome indicators was prepared, including: 1. Prevalence of active syphilis, 2. Prevalence of Human Papillomavirus (HPV) (high risk and low risk subtypes), 3. Prevalence of Neisseria Gonorrhoeae (NG), 4. Prevalence of Chlamydia Trachomatis (CT), 5. Knowledge of HIV, 6. Using condom during the last sex act with non-transactional and transactional male sex partners to prevent HIV and STIs, 7. FSW attended at least four antenatal care visits during their last pregnancy, and 8. Births in FSW attended by skilled birth attendant.

As soon as the list of outcome variables was finalized for the cross-sectional survey with FSW, we searched for data values for above-mentioned indicators from published literature and reports, to facilitate our sample size calculation. For each indicator, the sample size was calculated separately using standard formula-1 [39] denoted by n1 mentioned below with 1.4%−5.0% precision, 95% confidence interval (CI) and design effect of 1.489. Thereafter, sample sizes were adjusted for finite population correction (FPC) using formula-2 [40,41] denoted by n2 below and then inflated by 5% to adjust for refusal or non-response during face-to-face interview, denoted by n3 below. While applying FPC with formula-2 below, data on the population size of FSW were extracted from the national size estimation report of Bangladesh conducted in 2015 [36]. At the time of developing the protocol in 2022, the latest size estimate for FSW was only available until 2015. Notably, the last size estimation of FSW and other key populations at risk of HIV was published in 2023 by the Government of Bangladesh [26]. Finally, the highest values from the calculated sample sizes were chosen for first-round survey, as shown in Table 1. Thus, the maximum sample size for FSW was 513. The formulas used to calculate sample size at the first round of SRHR surveillance for FSW are given below:

**Table 1:** Sample size calculation.

SL#	Indicators	Values of the indicators (%)	Precision	Calculated sample size n1	Calculated sample size n2	Calculates sample size n3
	***Biological***					
1.	Prevalence of active syphilis	1.5 [42]	1.5%	375	339	356
2.	Prevalence of HPV (Cervical)	HPV 16: 10.1 HPV 18: 5.4 HPV 6: 3.6 [43]	3.5% 3.0% 2.0%	423 324 496	377 297 435	396 311 456
3.	Prevalence of *Neisseria Gonorrhea*	1.4 (Cervical) [44]	1.4%	402	361	379
4.	Prevalence of *Chlamydia trachomatis*	6.3 (Cervical) [44]	3.0%	375	339	356
	***Behavioral***					
5.	Comprehensive knowledge of HIV^§^	26.4 [45]	5.0%	444	394	414
6.	Used condom in the last sex act with a male sex partner	78.7 [45]	5.0%	383	345	363
7.	Percentage of FSW attended at least four antenatal care visits during pregnancy	27.7 [46]	5.0%	458	405	425
8.	Percentage of births from FSW attended by skilled birth attendant	54.1 [46]	5.0%	568	489	**513**

^§^This indicator was computed by correct answers to five questions [47].

Formula-1:


$$n_{2}=\frac{n_{1}}{1+\frac{n_{1}}{N}}$$

In the above equation:

n1 = Calculated sample size

D = Design effect

p = Estimated percentage points of the indicators at the time from which the data were extracted to conduct the first round of SRHR surveillance

q = 1-p

Zα/2 = Two tailed Z-score corresponding to the desired level of significance = 1.96 (with 95% CI)

d = Desired level of precision

Formula-2:


$$n_{\text{2}}=\frac{n_{\text{1}}}{\text{1}+\frac{n_{\text{1}}}{N}}$$

n1 = Calculated sample size without FPC

n2 = Calculated sample size after adjusting for FPC

N = Size of the FSW in Jashore

n3 = n2 x 1.05 (Calculated sample size after FPC and 5% adjusting for refusal or non-response during face-to-face interview)

1. Can people reduce their risk of HIV by using a condom correctly and consistently in any type of sex? (‘Yes’ was considered as correct answer)
2. Can people reduce their risk of HIV by avoiding sex with multiple partners? (‘Yes’ was considered as correct answer)
3. Can a person get HIV through mosquito bite? (‘No’ was considered as correct answer)
4. Can a person get HIV by sharing a meal with someone who is HIV infected? (‘No’ was considered as correct answer)
5. Can you tell by looking at someone whether s/he is infected with HIV? (‘No’ was considered as correct answer)

### Sampling methods and procedures

To conduct the first-round survey among FSW, we adopted a two-stage cluster sampling technique, known as time location sampling (TLS) [14,48]. In the first stage of sampling, a social mapping exercise was conducted to identify ‘cruising spots’ or primary sampling units (PSUs) where FSW were available during a particular time window, e.g., 6 PM to 10 PM. This enabled us to create a sampling frame of spots, thus allowing a random sample of spots and individuals. For brothel-based FSW, a spot refers to a specific room used in a brothel for selling sex; for street-based FSW, a specific location (such as, railway station, in-front of cinema hall, amusement park, etc.) where at least 3 FSW were found in a specific time frame who sell sex; for residence-based FSW, a specific house where at least 5 FSW were found in a specific time frame who sell sex; and for hotel-based FSW, a residential hotel where at least 5 FSW were found in a specific time frame who sell sex [14]. The timing of the spots was determined by visiting the spots beforehand along with secondary sources such as gatekeepers at the spots, service providers for FSW from the drop-in centers (DICs), community-based organizations (CBOs), and self-help groups (SHGs). The social mapping exercise in Jashore was conducted between July-August 2022. Mapping data were checked for inconsistencies and then entered in Excel. Finally, a cleaned version of mapping data file in Excel was prepared for the next step of sampling. The mapping data showed that the total number of FSW from all groups was 334, which was less than the calculated target sample size 513. Therefore, in the second stage of sampling a ‘take-all’ sampling was adopted by visiting as many spots as possible. Since the proportionate breakdown of the achieved sample size of the types of FSW was uneven (street FSW = 135, hotel FSW = 50, residence FSW = 75 and brothel FSW = 67), this analysis aggregated all types of FSW within a single group. This is to be noted that there are three sampling techniques to study hard-to-reach populations (e.g., FSW) such as snowball sampling, time location sampling (TLS), and respondent-driven sampling (RDS) [49]. Here, snowball is a non-probability sampling and TLS and RDS are probability sampling [50]. Since, the FSW in our study congregate in cruising spots that are identifiable and accessible, and a sampling frame can be made, we used (TLS) [51].

### Definition of the outcome variable in the analysis

The key outcome variable for this analysis was whether a participant has ever experienced an unintended pregnancy. If an FSW experienced unintended pregnancy at least once in their lifetime, they were categorized as ‘yes’ (coded = 1). Otherwise, they were considered as ‘no’ (coded = 0). FSW who reported never being pregnant (N = 20) were excluded from the analysis. Hence, the final analysis was conducted among those who reported a pregnancy at least once in their lifetime (N = 307; out of 327). Additionally, to facilitate a better understanding of the population characteristics, information on the outcome of the last unintended pregnancy was collected to understand whether the pregnancy resulted in live birth or an abortion. In the cases where the outcome was abortion, further information was collected on whether it was spontaneous or induced abortion.

### List of independent variables in the analysis

Based on programmatic knowledge and literature review, various independent variables were chosen and used for this analysis. These included:

- Duration of stay in Jashore (≤5 years; 6–10 years; > 10 years; outside from Jashore),
- Main source of income (other than sex work; sex work) in the last month,
- Years of schooling (no education; 1–5; ≥ 6),
- Age (15–24; 25–34; ≥ 35) in years,
- Marital status (currently married; currently unmarried),
- Income (≤10,000; > 10,000) in BDT in the last month,
- Age at first marriage (≤12; 13–15; ≥ 16) in years,
- Age at first sex (≤12; 13–15; ≥ 16) in years,
- Duration of selling sex (≤3; > 3) in years,
- Sexualized drug use during the last one year; In this study, sexualized drug use refers to any illicit drugs (i.e., cannabis, methamphetamine (locally known as yaba), alcohol, Tari/locally made alcohol, injection of opiate-based drugs, phensedyl, and pink pill) used during or before any planned sexual act primarily to facilitate, enhance, sustain or disinhibit a sexual experience [52].
- Sexual experience with non-transactional male sex partner ever in their lifetime,
- Ever experienced rape in their lifetime,
- Experience of physical violence during the last one year,
- Self-reported depression during the last one year,
- Self-reported anxiety during the last one year,
- Self-reported stress during the last one year,
- Self-reported mental health problems during the last one year (yes = self-reported depression, or anxiety, or stress; no = none),
- SRHR service uptake status during the last one year,
- Any self-reported symptoms of STI during the last one year, and
- Adequacy of SRHR knowledge;.
- Adequate SRHR knowledge was defined as a composite indicator that appended the answers to 24 knowledge questions. Initially, nine questions were used to assess knowledge of STI symptoms, treatments, prevention, and misconceptions; five questions evaluated reproductive physiology; three questions assessed knowledge of at least three contemporary methods of contraception; five questions evaluated knowledge of HIV; two questions evaluated sexual rights; and two additional questions evaluated reproductive rights. After that, ‘1’ was assigned to all correctly answered questions and ‘0’ to all incorrect answers. Responses from all the knowledge-related questions were summed up for an overall composite score of SRHR. Finally, the overall SRHR knowledge was classified those who obtained more than two-third of the knowledge scores based on literature evidence [53], which was considered above 18 in this analysis.

### Statistical analysis

Percentage points were used to report categorical variables along with 95% confidence intervals (CI). All respondents’ characteristics were compared between two groups of FSW (unintended and intended) using Chi-square statistic [54]. All comparisons were considered significant when p < 0.05. Furthermore, to identify the factors having unadjusted association with unintended pregnancy, bivariate analysis was conducted via univariate logistic regression modelling [55,56]. Finally, the adjusted association of factors associated with the experience of unintended pregnancy was measured using multiple logistic regression modelling [55,56]. Unadjusted association was expressed in terms of unadjusted odds ratio (UOR) and the adjusted association was articulated by adjusted odds ratio (AOR). Factors significant at 10% level in the bivariate analysis were included in the multiple logistic regression model [55,57,58]. Before running the multivariate analysis, to detect multi-collinearity, pair-wise correlation (r < 0.5), and variance inflation factor (VIF < 2) was checked among the significant factors in the bivariate analysis [55,59–61]. Clustering of observations was incorporated throughout the analysis. Hosmer–Lemeshow goodness of fit was reported as a measure of model fit [62]. In this analysis, all data were entered by Epi-Info 7.5.2. Before analysis, data were cleaned utilizing MS-Excel, and statistical analyses were conducted using STATA-17.

## Results

### Socio-demographic and other characteristics

The socio-demographic and other characteristics of ever-pregnant FSW in Jashore are presented in Table 2. Of 307 FSW, the majority were living in Jashore for more than ten years (76.9%; 95% CI: 66.9–84.5). They mainly earned from sex work (85.0%; 95% CI: 79.1–89.5) within the last one month preceding the interview. Over a third of the FSW had no formal education (35.5%; 95% CI: 29.8–41.7). FSW were mostly between 25–34 years of age (41.0%; 95% CI: 34.1–48.3), and majority had a monthly income of less than or equal to 10,000 BDT or approximately 83 USD (1 USD = 120 BDT [63]).

**Table 2:** Socio-demographic and other characteristics of the FSW in Jashore.

Variables	Total
	n, Col%, (95% CI)
	(N = 307)
**Socio demographic**	
Duration of stay in Jashore	
≤5 years	34, 11.1 (5.5-21.1)
6-10 years	22, 7.2 (4.5-11.2)
>10 years	236, 76.9 (66.9-84.5)
Outside form Jashore^‡^	15, 4.9 (1.8-12.9)
Main source of income during the last one month	
Other than sex work	46, 15.0 (10.5-20.9)
Sex work	261, 85.0 (79.1-89.5)
Years of schooling	
No education	109, 35.5 (29.8-41.7)
1-5	92, 30.0 (25.0-35.5)
≥6	106, 34.5 (28.2-41.5)
Age (in years)	
15-24	62, 20.2 (14.3-27.7)
25-34	126, 41.0 (34.1-48.3)
≥35^§^	119, 38.8 (29.9-48.5)
Income during the last month (in BDT)	
≤10,000	196, 63.8 (50.8-75.1)
>10,000	111, 36.2 (24.9-49.2)
Marital status	
Currently married	154, 50.2 (40.6-59.7)
Currently unmarried^¶^	153, 49.8 (40.3-59.4)
**Reproductive health**	
Age at first marriage (in years) (Denominator is those who were currently married/ divorced/ widow/ separated)	
≤12	N = 296 58, 19.6 (15.5-24.5)
13-15	149, 50.3 (46.0-54.7)
≥16	89, 30.1 (25.2-35.4)
Age at first sex (in years)	
≤12	69, 22.5 (18.7-26.8)
13-15	163, 53.1 (48.7-57.4)
≥16	75, 24.4 (20.8-28.5)
**Behavioral characteristics**	
Duration of selling sex (in years)	
≤3	N = 303^Ѳ^ 116, 38.3 (29.3-48.1)
>3	187, 61.7 (51.9-70.7)
Sexualized drug use during the last one year	
Yes	58, 18.9 (13.5-25.8)
No	249, 81.1 (74.2-86.5)
Sexual experience with non-transactional male sex partner ever in their lifetime	
Yes	287, 93.5 (89.5-96.0)
No	20, 6.5 (4.0-10.5)
**Violence (sexual/physical)**	
Ever experienced rape in their lifetime	
Yes	87, 28.3 (22.8-34.7)
No	220, 71.7 (65.3-77.2)
Faced physical violence during the last one year	
Yes	41, 13.4 (9.4-18.6)
No	266, 86.6 (81.4-90.6)
**Self-reported mental health**	
Self-reported depression during the last one year	
Yes	178, 58.0 (52.2-63.5)
No	129, 42.0 (36.5-47.8)
Self-reported anxiety during the last one year	
Yes	212, 69.1 (63.7-74.0)
No	95, 30.9 (26.0-36.3)
Self-reported stress during the last one year	
Yes	190, 61.9 (54.9-69.4)
No	117, 38.1 (31.6-45.1)
Self-reported mental health problem during the last one year (depression/anxiety/stress)	
Yes	223, 72.6 (67.8-77.0)
No	84, 27.4 (23.0-32.2)
**SRHR service uptake**	
SRHR service uptake status during the last one year	
Yes	306, 99.7 (97.9-99.9)
No	1, 0.3 (0.1-2.1)
**STI symptoms**	
STI symptoms during the last one year	
Yes	216, 70.4 (63.7-76.2)
No	91, 29.6 (23.8-36.3)
**SRHR knowledge**	
Adequate knowledge of SRHR	
Yes	136, 44.3 (35.2-53.8)
No	171, 55.7 (46.2-61.8)

Col% = column percentage.

§The maximum reported age was 54 years; only one FSW was found at this age.

¶Currently unmarried includes unmarried/ divorced/ widow/ separated.

Ѳ4 FSW did not respond.

‡These FSW live in adjacent districts from Jashore, and they come to Jashore to sell sex and go back home in the evening.

About half of the FSW were married at the time of interview (50.2%; 95% CI: 40.6–59.7), and the age of their first marriage was between 13–15 years (50.3%; 95% CI: 46.0–54.7). Majority of the FSW in Jashore were selling sex for more than three years (61.7%; 95% CI: 51.9–70.7). More than a quarter of the FSW experienced rape at some point during their lifetime (28.3%; 95% CI: 22.8–34.7). Almost three-quarter of them had self-reported mental health problems (72.6%; 95% CI: 67.8–77.0). Except one, all FSW reported availing of SRHR services during the last one year. Among the FSW, 70.4% (95% CI: 63.7–76.2) reported STI symptoms within the last one year and 44.3% (95% CI: 35.2–53.8) of the FSW had adequate knowledge of SRHR.

### History of unintended pregnancy

**Fig 1:**
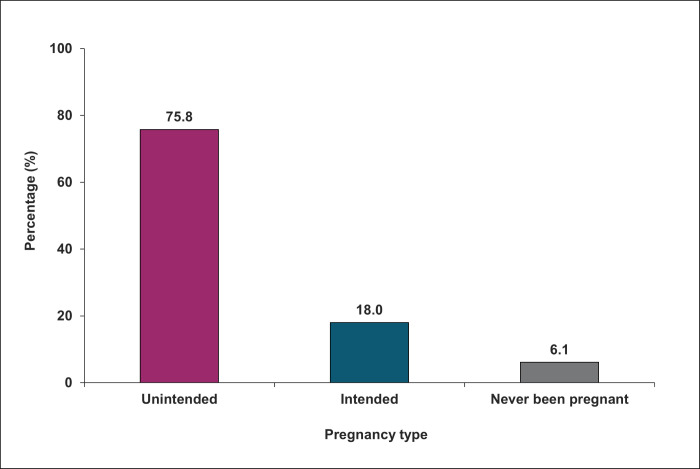
Prevalence of unintended pregnancy among FSW in their lifetime (N = 327).

**Fig 2:**
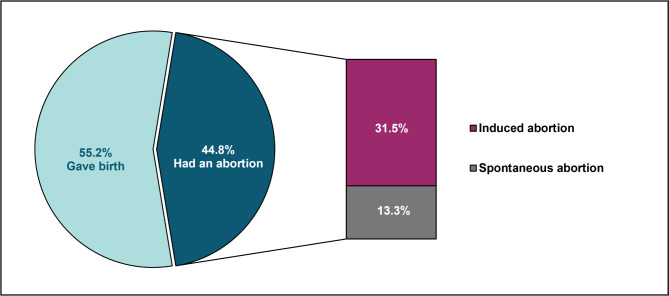
Outcome of the last unintended pregnancy (N = 248).

The prevalence of unintended pregnancy among 327 FSW was 75.8% (95% CI: 71.0–80.1, N = 307) (Fig 1). Outcomes of the last unintended pregnancy are in Fig 2. Findings indicated that a substantial proportion of the unintended pregnancies ended up in abortion during their most recent (44.8%; 95% CI:) pregnancy. Further analysis depicted that, among the 44.8% who experienced abortion during the most recent unintended pregnancy, 31.5% (95% CI: 25.6–37.9) were induced and 13.3% (95% CI: 8.8–19.7) were spontaneous abortions (Fig 2).

### Comparison of characteristics between FSW who had ever experienced unintended pregnancy and those who did not

According to the findings of Table 3, more FSW involved with sex work who had ever experienced unintended pregnancy in their lifetime compared to FSW who reported intended pregnancy (87.1% vs. 76.3%, p = 0.038). More FSW had 1–5 years of schooling who reported had intended pregnancy ever in their lifetime than those who had ever experienced unintended pregnancy (37.3% vs. 28.2%, p = 0.032). More FSW reported experienced rape in their lifetime who had ever experienced unintended pregnancy compared to FSW who reported intended pregnancy (31.0% vs. 16.9%, p = 0.015). More FSW had suffered from anxiety during the last one year who had ever experienced unintended pregnancy than those who reported intended pregnancy (72.2% vs. 55.9%, p = 0.022). More FSW had suffered mental health problem during the last one year who had ever experienced unintended pregnancy compared to FSW who reported intended pregnancy (76.2% vs. 57.6%, p = 0.017).

**Table 3:** Comparison of characteristics of the respondents in the context of their intended and unintended pregnancy status.

Variables	Unintended	Intended	Comparison
	n, Col%, (95% CI)	n, Col%, (95% CI)	p-value
	(N = 248)	(N = 59)	
**Socio demographic**			
Duration of stay in Jashore			
≤5 years	27, 10.9 (4.5-23.8)	7, 11.9 (6.3-21.3)	0.865
6-10 years	16, 6.5 (3.4-12.0)	6, 10.2 (5.1-19.2)	0.373
>10 years	194, 78.2 (64.9-87.4)	42, 71.2 (61.6-79.2)	0.373
Outside form Jashore	11, 4.4 (1.5-12.2)	4, 6.8 (2.1-20.1)	0.304
Main source of income during the last one month			
Other than sex work	32, 12.9 (8.7-18.7)	14, 23.7 (14.1-37.1)	0.038
Sex work	216, 87.1 (81.3-91.3)	45, 76.3 (62.9-85.9)	0.038
Years of schooling			
No education	92, 37.1 (30.8-43.8)	17, 28.8 (20-39.6)	0.128
1-5	70, 28.2 (23.4-33.7)	22, 37.3 (27.7-48.0)	0.032
≥6	86, 34.7 (27.4-42.8)	20, 33.9 (24.7-44.5)	0.897
Age (in years)			
15-24	52, 21.0 (15.0-28.6)	10, 16.9 (8.0-32.3)	0.521
25-34	99, 39.9 (32.8-47.5)	27, 45.8 (33.8-58.2)	0.332
≥35^§^	97, 39.1 (30.8-48.1)	22, 37.3 (22.6-54.8)	0.777
Income during the last month (in BDT)			
≤10,000	156, 62.9 (49.7-74.4)	40, 67.8 (51.6-80.6)	0.389
>10,000	92, 37.1 (25.6-50.3)	19, 32.2 (19.4-48.4)	0.389
Marital status			
Currently married	123, 49.6 (39.3-59.9)	31, 52.5 (38.4-66.3)	0.696
Currently unmarried^¶^	125, 50.4 (40.1-60.7)	28, 47.5 (33.7-61.6)	0.696
**Reproductive health**			
Age at first marriage (in years) (Denominator is those who were currently married/ divorced/ widow/ separated)			
≤12	N = 238 46, 19.3 (14.3-25.7)	N = 58 12, 20.7 (12.1-33.1)	0.833
13-15	120, 50.4 (44.4-56.5)	29, 50 (35.1-64.9)	0.965
≥16	72, 30.3 (24.7-36.5)	17, 29.3 (19.9-40.9)	0.880
Age at first sex (in years)			
≤12	56, 22.6 (18.4-27.4)	13, 22.0 (13.5-33.9)	0.924
13-15	133, 53.6 (48.9-58.3)	30, 50.8 (37.8-63.8)	0.701
≥16	59, 23.8 (19.9-28.2)	16, 27.1 (17.6-39.4)	0.568
**Behavioral characteristics**			
Duration of selling sex (in years)			
≤3	N = 245 98, 40 (30.7-50.0)	N = 58 18, 31.0 (19.5-45.5)	0.159
>3	147, 60 (50.0-69.3)	40, 69.0 (54.5-80.5)	0.159
Sexualized drug use during the last one year			
Yes	51, 20.6 (14.7-28.0)	7, 11.9 (6.1-21.8)	0.055
No	197, 79.4 (72.0-85.3)	52, 88.1 (78.2-93.9)	0.055
Sexual experience with non-transactional male sex partner ever in their lifetime			
Yes	235, 94.8 (89.1-97.6)	52, 88.1 (81.8-92.5)	0.070
No	13, 5.2 (2.4-10.9)	7, 11.9 (7.5-18.2)	0.070
**Violence (sexual/physical)**			
Ever experienced rape in their lifetime			
Yes	77, 31.0 (25.2-37.6)	10, 16.9 (9.5-28.4)	0.015
No	171, 69.0 (62.4-74.8)	49, 83.1 (71.6-90.5)	0.015
Faced physical violence during the last one year			
Yes	34, 13.7 (9.9-18.7)	7, 11.9 (5.4-24.1)	0.670
No	214, 86.3 (81.3-90.1)	52, 88.1 (75.9-94.6)	0.670
**Self-reported mental health**			
Self-reported depression during the last one year			
Yes	150, 60.5 (60.5-54.6)	28, 47.5 (33.2-62.2)	0.101
No	98, 39.5 (33.9-45.4)	31, 52.5 (37.8-66.8)	0.101
Self-reported anxiety during the last one year			
Yes	179, 72.2 (65.9-77.7)	33, 55.9 (43.1-68.0)	0.022
No	69, 27.8 (22.3-34.1)	26, 44.1 (32.0-56.9)	0.022
Self-reported stress during the last one year			
Yes	160, 64.5 (57.6-70.8)	30, 50.8 (36.0-65.5)	0.065
No	88, 35.5 (29.2-42.4)	29, 49.2 (34.5-64.0)	0.065
Self-reported mental health problem during the last one year			
Yes	189, 76.2 (69.9-81.5)	34, 57.6 (44.3-69.9)	0.017
No	59, 23.8 (18.5-30.1)	25, 42.4 (30.1-55.7)	0.017
**SRHR service uptake**			
SRHR service uptake status during the last one year			
Yes	247, 99.6 (97.3-99.9)	59, 100.0 (93.9-100.0)	0.635
No	1, 0.4 (0.1-2.7)	0	0.635
**STI symptoms**			
STI symptoms during the last one year			
Yes	175, 70.6 (63.2-77.0)	41, 69.5 (58.7-78.5)	0.842
No	73, 29.4 (23.0-36.8)	18, 30.5 (21.5-41.3)	0.842
**SRHR knowledge**			
Adequate knowledge of SRHR			
Yes	111, 44.8 (35.9-54.0)	25, 42.4 (28.1-58.1)	0.110
No	137, 55.2 (46.0-64.1)	34, 57.6 (41.9-71.9)	0.110

Col% = column percentage.

§The maximum reported age was 54 years; only 1 FSW was found at this age.

¶Currently unmarried includes unmarried/ divorced/ widow/ separated.

### Factors associated with unintended pregnancy

The results of bivariate analysis depicted significant associations between unintended pregnancy and their main source of income in the last one month, sexualized drug use over the last one year, history of non-transactional sex with male sex partners throughout their lifetime, experience of rape, self-reported mental health problems during the last one year, which were significant at p < 0.10 (as shown in Table 4).

**Table 4:** Factors associated with unintended pregnancy.

Factors	Bivariate analysis				Multivariate analysis	
	Unintended	Intended	UOR (95% CI)	p-value	AOR (95%CI)	p-value
	n/N (Row%)	n/N (Row%)				
Duration of stay in Jashore						
≤5 years (RC)	27/34 (79.4%)	7/34 (20.6%)	1.0	--	--	**--**
6-10 years	16/22 (72.7%)	6/22 (27.3%)	0.7 (0.2-2.0)	0.483		
>10 years	194/236 (82.2%)	42/236 (17.8%)	1.2 (0.4-4.0)	0.763		
Outside form Jashore	11/15 (73.3%)	4/15 (26.7%)	0.7 (0.2-3.3)	0.661		
Main source of income during the last one month						
Other than sex work (RC)	32/46 (69.6%)	14/46 (30.4%)	1.0	--	1.0	--
Sex work	216/261 (82.8%)	45/261 (17.2%)	2.1 (1.0-4.3)	0.041	2.0 (0.9-4.3)	0.089
Years of schooling						
No education	92/109 (84.4%)	17/109 (15.6%)	1.3 (0.7-2.4)	0.469		
1-5	70/92 (76.1%)	22/92 (23.9%)	0.7 (0.4-1.3)	0.259		
≥6 (RC)	86/106 (81.1%)	20/106 (18.9%)	1.0	--	--	--
Age (in years)						
15-24	52/62 (83.9%)	10/62 (16.1%)	1.2 (0.5-2.9)	0.716		
25-34	99/126 (78.6%)	27/126 (21.4%)	0.8 (0.5-1.4)	0.494		
≥35 (RC)	97/119 (81.5%)	22/119 (18.5%)	1.0	--	--	--
Income during the last one month (in BDT)						
≤10,000 (RC)	156/196 (79.6%)	40/196 (20.4%)	1.0	--	--	--
>10,000	92/111 (82.9%)	19/111 (17.1%)	1.2 (0.8-2.1)	0.390		
Marital status						
Currently married (RC)	123/154 (79.9%)	31/154 (20.1%)	1.0	--	--	--
Currently unmarried	125/153 (81.7%)	28/153 (18.3%)	1.1 (0.6-2.1)	0.696		
Age at first marriage (in years) (Denominator is those who were currently married/ divorced/ widow/ separated)						
≤12 (RC)	46/58 (79.3%)	12/58 (20.7%)	1.0	--	--	--
13-15	120/149 (80.5%)	29/149 (19.5%)	1.1 (0.4-2.8)	0.874		
≥16	72/89 (80.9%)	17/89 (19.1%)	1.1 (0.5-2.3)	0.786		
Age at first sex (in years)						
≤12 (RC)	56/69 (81.2%)	13/69 (18.8%)	1.0	--	--	--
13-15	133/163 (81.6%)	30/163 (18.4%)	1.0 (0.5-2.1)	0.936		
≥16	59/75 (78.7%)	16/75 (21.3%)	0.9 (0.4-1.8)	0.681		
Duration of selling sex (in years)						
≤3	98/116 (84.5%)	18/116 (15.5%)	1.5 (0.9-2.6)	0.161		
>3 (RC)	147/187 (78.6%)	40/187 (21.4%)	1.0	--	--	--
Sexualized drug use during the last one year						
Yes	51/58 (87.9%)	7/58 (12.1%)	1.9 (1.0-3.8)	0.059	1.4 (0.8-2.7)	0.264
No (RC)	197/249 (79.1%)	52/249 (20.9%)	1.0	--	--	--
Sexual experience with non-transactional male sex partner ever in their lifetime						
Yes	235/287 (81.9%)	52/287 (18.1%)	2.4 (0.9-6.6)	0.078	2.4 (1.1-5.3)	0.036
No (RC)	13/20 (65.0%)	7/20 (35.0%)	1.0	--	1.0	--
Ever experienced rape in their lifetime						
Yes	77/87 (88.5%)	10/87 (11.5%)	2.2 (1.2-4.2)	0.017	2.0 (1.0-3.8)	0.037
No (RC)	171/220 (77.7%)	49/220 (22.3%)	1.0	--	1.0	--
Faced physical violence during the last one year						
Yes	34/41 (82.9%)	7/41 (17.1%)	1.2 (0.5-2.6)	0.671		
No (RC)	214/266 (80.5%)	52/266 (19.5%)	1.0	--	--	--
Self-reported mental health problem during the last one year						
Yes	189/223 (84.8%)	34/223 (15.2%)	2.4 (1.2-4.8)	0.019	2.1 (1.0-4.2)	0.045
No (RC)	59/84 (70.2%)	25/84 (29.8%)	1.0	--	1.0	--
STI symptoms during the last one year						
Yes	175/216 (81.0%)	41/216 (19.0%)	1.1 (0.6-1.8)	0.842		
No (RC)	73/91 (80.2%)	18/91 (19.8%)	1.0	--	--	--
Adequate knowledge of SRHR						
Yes	111/136 (81.6%)	25/136 (18.4%)	1.1 (0.7-1.9)	0.713		
No (RC)	137/171 (80.1%)	34/171 (19.9%)	1.0	--	--	--

Number of observations in the multivariate analysis, N = 307.

Hosmer-Lemeshow goodness = 0.947 (p > 0.05 means good model fit).

RC = reference category; UOR = Unadjusted odds ratio; AOR = Adjusted odds ratio; CI = Confidence interval.

Three out of five variables identified as significant in the bivariate analysis also demonstrated significance in the multivariate analysis (p < 0.05), i.e., lifetime sexual experience with non-transactional male sex partner, lifetime experience of rape, and self-reported mental health problem during the last one year (as shown in Table 4). Findings depicted that, participants who had sexual experiences with non-transactional male sex partner ever in their lifetime were 2.4 times (95% CI: 1.1–5.3, p = 0.036) more likely to have ever experienced unintended pregnancy than those who never had sex with a non-transactional male sex partner. Additionally, those who reported experience rape in their lifetime were 2.0 times (95% CI: 1.0–3.8, p = 0.037) more likely to had ever experienced unintended pregnancy than those did not report to have experienced rape. Furthermore, those who self-reported mental health problems were 2.1 times (95% CI: 1.0–4.2, p = 0.045) more likely to had ever experienced unintended pregnancy than those who did not self-report any mental health problem during the last one year.

## Discussion

The results of our study show the prevalence and associated factors of unintended pregnancies among FSW in Jashore. The study documented a substantial proportion of unintended pregnancies, of which almost half ended up in induced or spontaneous abortions. Furthermore, sexual intercourse with a non-transactional male sex partner, experienced rape in their lifetime, and self-reported mental health problems in the last one year were identified as the key factors associated with unintended pregnancies.

Relative to the high prevalence ever experiencing unintended pregnancy revealed in our study, studies in other countries like Afghanistan, Swaziland, Kenya, Ethiopia, showed lower prevalence of unintended pregnancy experienced by the FSW (such as 28.6%, 36.9%, 49.0%, and 52.0%, respectively) [64–68]. Whereas, a study conducted among FSW in Uganda reported that 23.9% were ambivalent about their pregnancies, and 65.1% did not plan their pregnancy, indicating unintended pregnancy to be at 89.0%, which was higher than our study findings [69]. Another study highlighted that Bangladesh has the highest recorded prevalence of unintended pregnancy (28.4%) among the general female population in the South Asian region [2]. This indicates that the unintended pregnancy rate among FSW is nearly 2.7 times higher, according to our study, thus showing their relative vulnerability compared to their general counterparts. From the above review, we can say that the prevalence of the experience of unintended pregnancy among FSW in Jashore Bangladesh is comparatively higher. In Bangladesh FSW mainly relies on condom as primary contraceptive method, where in most cases the usage remains inconsistent, while fear of disclosure reduces uptake of family planning [11,70]. High levels of stigma and criminalization further discourage regular engagement with reproductive health services [70]. Frequent disruptions in condom use due to gender based violence or power imbalances may contribute to and explain the high unintended pregnancy rates found in our study [11,70].

Unintended pregnancy that leads to spontaneous or induced abortion is another occupational hazard faced by FSW [71]. Our study revealed that approximately one-third of FSW reported induced abortion, mirroring the worldwide occurrence of 37.7% among FSW, as shown in a recent systematic review and meta-analysis of 48 studies from 34 countries by Khezri and colleagues [71]. This emphasizes the need for ensuring availability and accessibility of effective contraception methods, along with safe abortion care by trained healthcare providers [71]. Similarly, a cross-sectional study among FSW in Bangladesh by Wahed and colleagues (2017) reported that abortion was primarily managed through menstrual regulation using manual vacuum aspiration, dilation and curettage, or medication [46]. Wahed and her colleagues also reported that, 57.5% of those who had an abortion experienced post-abortion complications such as excessive bleeding, lower abdominal pain, severe weakness, blurry vision, or incomplete abortion [46]. Alarmingly, about 2 out of 5 FSW either sought care from unqualified providers or did not seek treatment at all [46]. Based on the findings derived from the multivariate analysis, through the lens of the socio-ecological model highlights the multi-layered determinants (i.e., individual, interpersonal, community/organizational, and structural) shaping the experience of unintended pregnancies among FSW[72,73]. Although individual factors are often associated with unintended pregnancies, these findings need to be understood as part of structurally constrained environments characterized by stigma, criminalization, gender-based violence and compromised reproductive autonomy.

At the individual level, our multivariate analysis revealed that self-reported mental health problems were positively associated with unintended pregnancy. Notably, unintended pregnancy may have occurred any time before the survey, whereas self-reported mental health problems were reported within the previous year of the survey. Therefore, unintended pregnancy may have occurred before or after having a mental health problem. Likewise, various studies portrayed a significant positive association between mental health problems and unintended pregnancy resulting from mental health distress [74–76]. Several reasons were identified such as: individuals with higher burden of mental health problems are more likely to engage in sex, and less likely to care about the consequences of unprotected sex, as well as their ambivalence towards family planning [74–76]. On the other hand, different retrospective case control and cohort studies portrayed that unintended pregnancies could contribute to mental health problems among women [77–79]. Beyond behavioral disinhibition, mental health problems may elevate unintended pregnancy risk through trauma-related symptoms, e.g., decreased self-efficacy, impaired decision-making, reduced negotiation powers for contraceptive, and challenges in maintaining consistent contraceptive use [80–82].

Although it is challenging to establish a temporal relationship between mental health problems and unintended pregnancies from cross-sectional data, a longitudinal study determined that mental health problems could lead to unintended pregnancies [76]. Thus, the presence of mental health problems places female sex workers at an elevated risk of unintended pregnancies, necessitating greater attention from policymakers. However, future research initiatives could entail assessing their mental health burden through validated psychometric tools. This could subsequently inform evidence-based mental health interventions. These interventions could entail the capacity strengthening of peer educators to deliver behavior change communication education, and teach basic coping mechanisms for managing distress. Moreover, embedding psychosocial first aid into the routine HIV and STI screening sessions in the existing FSW interventions will enable FSW to be screened with simple tools, and then subsequently offered group psychoeducation sessions or one-to-one support by trained existing peer outreach workers that will have no cost implications. Notably, mental health problems among FSW do not occur in isolation but are influenced by chronic exposure to structural violence, societal stigma, unsolicited policing, and economic precarity. All of these factors are structurally embedded in sex work contexts [81,83].

At the interpersonal level, respondents who reported sexual experiences with a non-transactional male sex partner were found to have higher odds of unintended pregnancies in this study. Similar scenarios were reflected in the existing literature, where having a steady non-transactional male sex partner was a major risk factor of unintended pregnancy [66,68,84]. Literature has attributed this to higher trust and a deeper relationship with the non-transactional male sex partner, often accompanied by dependency within these relationships, compared to transactional sex partners [68,85,86]. This is also coupled with overarching gender power dynamics which expect women to submit to their partners’ expectations, or else they face retaliation, especially in patriarchally dominated South Asian societies [87–89]. As a result, individuals may manifest a tendency to differentiate between their non-transactional partner from transactional ones through unprotected sex, thus potentially contributing to unintended pregnancies [68,85]. These dynamics operate through reduced condom negotiation powers, fear of violence or conflict, and normalization of unprotected sex within intimate relationships, thus elevating likelihood of unintended pregnancy. This reflects not only individual choices but constrained reproductive decision-making within unequal intimate partnerships. On this note, another study conducted in Dhaka, Bangladesh posited that FSW believed that insisting on using condoms with their husbands would constitute physical abuse [70]. FSWs’ husbands were also found to refuse condom use, thus driving unprotected sex and, eventually, unintended pregnancies [70]. These upstream determinants of structural violence within patriarchal societies often led to compromised reproductive autonomy, thus elevating risks of unintended pregnancies [89,90].

To alleviate this complexity, various studies suggested dual protection methods including a non-barrier or other highly effective contraception method (such as pills/injectable/implants/intrauterine device) alongside condom use [13,84,91,92]. However, this presents programmatic challenges from the perspective of HIV infection, as non-barrier contraception use could perpetuate unprotected sex [91,92]. Thus, it is crucial to integrate condom use and non-barrier contraception within the existing FSW interventions both in Bangladesh and other settings to simultaneously mitigate the risk of HIV and other STIs, alongside repeated unintended pregnancies [13,91]. In conjunction with supporting dual protection methods, non-transactional male sex partners need to be included in the counselling initiatives within existing FSW interventions for improving overall condom-use norms, condom-use negotiation, and self-efficacy [93]. Literature shows that alternatives such as multipurpose protection technologies (MPTs) (e.g., vaginal ring, dual prevention pill etc.) are under development to prevent unintended pregnancies, STIs and HIV simultaneously [94–97]. However, to implement this, several challenges need to be overcome such as funding and resource allocation, clinical trials, regulatory challenges, logistical complexity, high production cost, distribution, user acceptance and adherence, etc. [95,97]. Recent studies suggested that MPTs could be a promising and comprehensive solution to deal with unintended pregnancies, STIs and HIV at the same time [94–97].

At the community and organization levels, results from our multivariate analysis also found that respondents who experienced rape at some point in their lifetime had higher odds of unintended pregnancy. On a similar note, various literature from other countries showed that experience of rape within the broader umbrella of sexual violence was positively related to unintended pregnancy/unintended births among FSW, general women and adolescents in various parts of the world including Bangladesh [69,98–100]. A qualitative study in India revealed an underlying explanation that arguments and coercive sexual episodes could emerge if women fail to fulfil her husband’s sexual demands, thus resulting in compromised reproductive autonomy and, hence, unintended pregnancies [101]. Furthermore, sexual violence also contributes to the reduction of decision-making capacity among women about their SRH, thus compounding their risk of unprotected sex and unintended pregnancy [94,98,102,103]. Sexual violence increases the risk of unintended pregnancy risk through several pathways, including coerced unprotected sex, inability to refuse or negotiate contraception, trauma-related disengagement from SRH services and a fear of identity disclosure [89,90]. A study conducted in Dhaka, Bangladesh identified that 28.0% of the hotel-based FSW, and 54.0% of the street FSW were forced to have sex within the last one year of their data collection [70], which corroborates our finding that a considerable proportion of FSW are subject to rape. This calls for a possible integration of early detection of sexual violence and emergency support services with the existing family planning service by the government and private organizations [98,104]. Gender-based violence not only operates through episodic events of rape, but also overarching coercive dynamics that limit negotiation powers, normalize unprotected sex, and compromise reproductive decision-making.

These findings have illuminated critical insights about the burden and correlates of unintended pregnancies amongst a highly marginalized, vulnerable group. At the structural level, such findings are crucial for informing SRHR and women’s health interventions, especially since unintended pregnancies embody health, social and economic ramifications. Due to fear of disruptions in income generation and elevated exposure to violence, FSW are more likely to delay healthcare-seeking, which, when coupled with healthcare institutional barriers, could amplify their risk of adverse pregnancy outcomes [105–107]. Stigma within mainstream healthcare settings further compounds these risks, as FSW often avoid contraceptive and abortion services due to fears of discrimination, lack of scope for confidential services, and moral policing by healthcare providers [18,106,108]. In settings where safe abortion is financially or legally difficult to access, many FSW resort to unsafe methods, which could exacerbate their risk of hemorrhage, sepsis, uterine perforation, and long-term infertility [109,110]. Moreover, for FSW, the legal marginalization related to sex work and abortion creates compounded vulnerability, where seeking SRH-related care embodies socio-legal and economic risks. Additionally, the syndemic trauma of unintended pregnancy and unsafe abortion precipitates mental health conditions such as depression and anxiety, thus further lessening their ability to negotiate safer sex [111,112]. Our findings correspond to global evidence from Sub-Saharan African and South Asian regions, which demonstrate that unintended pregnancy among FSW is less driven by contraceptive ignorance and more by structural constraints, including stigma, violence and legal marginality [106,113]. These structural dynamics cumulatively reflect compromised reproductive autonomy, where women’s ability to decide their pregnancy conditions and outcomes are systematically undermined. These mechanisms are further intensified by chronic exposure to stigma, violence, and policing, all of which influence mental distress and reproductive vulnerability.

Addressing these health complexities is particularly crucial in the contexts where abortion is in a socio-legally precarious situation. For instance, in Bangladesh, abortion is criminalized under Section 312 of the Penal Code (1860), only permitted on the grounds of saving the pregnant mother’s life [114]. However, the Government of Bangladesh has authorized menstrual regulation up to 10–12 weeks after the missed period [115]. Despite this policy, stigma, socio-cultural taboos, and limited availability of trained healthcare providers, especially in rural areas, often lead women to self-terminate or delay care, thus exacerbating their risk of complications [116,117].

## Strengths, weaknesses, and challenges

This study has its own set of strengths, weaknesses, and challenges. Firstly, and most notably, this study demonstrated the success of the two-stage probability sampling approach which utilized FSW networks for the first time in Bangladesh, while conducting an SRHR surveillance among FSW in Jashore. Notably, the TLS strategy that we applied in our study is based on standard sample survey methods [118]. It has a validated theoretical basis therefore; this sampling strategy is able to fit models and has a wide range of statistical software to analyze data [118]. On the other hand, TLS has included FSW those who used to visit the cruising spots at the time of data collection and therefore, FSW who did not visit cruising spots or who were hidden at the time of data collection were missed [119].

There are some weaknesses in our study during data collection. Data were collected from a vulnerable population, involving personal and sensitive information, including whether an abortion was spontaneous or induced, in the context where the legality of abortion is ambiguous in Bangladesh. Consequently, this could potentially culminate in social desirability bias [120]. This was alleviated by: 1) ensuring that the information provided will be kept confidential and will solely be used for research, 2) asking questions in a non-judgmental and non-leading way, and 3) field-testing the questionnaire to identify and address possible biases [121]. Furthermore, the presence of recall periods for certain inquiries introduces the likelihood of recall bias [122]. This was mitigated through: 1) utilizing highly trained interviewers, 2) standardizing the survey questions in simple language, and 3) recalling information, calendars/timelines to help the respondents accurately recall events [123]. In this study, mental health problems were assessed with self-reported measures instead of validated psychometric instruments. Although the literature demonstrated its effectiveness and adaptability across various demographics, caution needs to be taken while interpreting the results as it may limit the reliability of this measure [124,125]. We acknowledge that it is difficult to measure whether mental health problems led to unintended pregnancies or vice versa due to the cross-sectional nature of our study. In our study, during mapping the number of FSW was less than the target sample size for two reasons. These were 1) due to urbanization, some FSW spots became defunct, thus scattering FSW to other obscure locations, 2) some FSW were highly mobile therefore, they frequently went to other districts, their native village, and India. We also could not achieve the target sample size because of three reasons, 1) most FSW mentioned they can earn a lot of money by selling sex which they have to compromise if they come to the data collection office to provide swabs, blood samples and behavioral information 2) in some hotels, FSW usually sell sex from 7 AM-6 PM, therefore they become tired and rush back to their residence; and 3) some FSW mentioned that they did not have any STIs therefore, they were not interested. It is worth mentioning that a smaller sample size compared to the target sample size may affect the precision of the estimates and therefore precautions should be taken in generalizing the findings [126]. However, this study still provides important evidence, generated through rigorous data collection and comprehensive efforts of data quality assurance, among a population that remains underrepresented in research.

The first challenge during data collection was that some FSW were reluctant to provide time to collect biological samples and behavioral information. During the process of obtaining consent/assent, data collectors explained to the participant the benefits of participating in this study such as opportunities for free testing and treatment of STIs, along with scope for free counselling on psychosocial/psychosexual/mental health from a psychologist in the surveillance team. The second challenge was that some FSW work during the weekdays, therefore the study team also collected behavioral information and biological samples during the weekend. The third challenge was that hotel managers of hotel-based FSW and house owners of residence-based FSW were reluctant to allow the team to collect samples and behavioral information from FSW. To enable a suitable environment, the study team convened several co-ordination meetings with hotel managers and house owners to explain the need and benefits of the study, to which they eventually agreed.

## Recommendations

The findings of the study could ultimately inform national SRHR program and refine the extant of HIV strategies for FSW and other underserved women. These results could inform changes to the Fifth National Strategic Plan for HIV and AIDS (2024–2028), the Ministry of Health and Family Welfare, the Government of Bangladesh [127] by recommending explicit inclusion of mental health indicators, and partner-inclusive approaches within existing FSW interventions. Detailed recommendations are described below:

1. To redesign FSW interventions to adapt dual protection, i.e., combining any female modern contraception such as pills/injectable/implants/intrauterine device (IUD) along with condoms to avoid not only the risk of repeated unintended pregnancies but also STIs and HIV [13,84,91,92,104].
2. Interventions also need to be initiated for the non-transactional male sex partners of the FSW to encourage condom-use during sex [86,93]. A few partner-inclusive interventions in South Asia demonstrated high feasibility, such as the Samvedana Plus trial in Karnataka, India. This model trained peer educators to convene counselling sessions with FSW and their intimate partners [128].
3. Ensure the reporting of sexual violence among FSW and providing immediate emergency support service (such as ensuring availability and accessibility to emergency contraceptives [68,104]) in the current family planning service of the government and private organizations [98].
4. Further research is needed to measure mental health using psychometric scales and its relationship with family planning and unintended pregnancies.

## Conclusion

The analysis of our study revealed a high prevalence of unintended pregnancies among FSW in Jashore, compounded by heightened risks of induced abortions. We identified specific factors, such as non-transactional male sexual partnerships, reported experienced rape, and self-reported mental health problems positively contributing to this issue. Without properly addressing these factors by strengthening policies and reproductive healthcare interventions, it would not be possible to improve the reproductive health of FSW. Moreover, these populations have historically been neglected in terms of other health conditions besides HIV and STIs, therefore FSW interventions need to assume a holistic approach in order to avert adverse reproductive health outcomes.

## Abbreviations

AIDS: Acquired Immunodeficiency Syndrome
AOR: Adjusted Odds Ratio
CBOs: Community-Based Organizations
CI: Confidence Intervals
CT: Chlamydia Trachomatis
DMA: Data Management Assistant
DMO: Data Management Officer
DICs: Drop-in Centers
FPC: Finite Population Correction
FRO: Field Research Officer,
FSW: Female Sex Workers
HIV: Human Immunodeficiency Virus
HPV: Human Papillomavirus,
IUD: Intrauterine Device
LMICs: Low-and Middle-Income Countries
MPTs: Multipurpose Prevention Technologies
NG: Neisseria Gonorrhoeae
PSUs: Primary Sampling Units
RI: Research Investigator
RTIs: Reproductive Tract Infections
SHGs: Self-Help Groups
SRH: Sexual and Reproductive Health
SRHR: Sexual and Reproductive Health and Rights
STIs: Sexually Transmitted Infections
TLS: Time Location Sampling
UOR: Unadjusted Odds Ratio.
